# *Prunella vulgaris* polysaccharide inhibits herpes simplex virus infection by blocking TLR-mediated NF-κB activation

**DOI:** 10.1186/s13020-023-00865-y

**Published:** 2024-01-08

**Authors:** Xuanlei Zhong, Yibo Zhang, Man Yuan, Lin Xu, Xiaomei Luo, Rong Wu, Zhichao Xi, Yang Li, Hongxi Xu

**Affiliations:** 1 School of Pharmacy, Shanghai University of Traditional Chinese Medicine, No. 1200, Cailun Road, Shanghai, 201203 China; 2 Engineering Research Center of Shanghai Colleges for TCM New Drug Discovery, Shanghai, China; 3 Shuguang Hospital, Shanghai University of Traditional Chinese Medicine, Shanghai, China

**Keywords:** Herpes simplex virus, *Prunella vulgaris* polysaccharide, TLR/NF-κB signalling pathway, Necroptosis

## Abstract

**Background:**

*Prunella vulgaris* polysaccharide extracted by hot water and 30% ethanol precipitation (PVE30) was reported to possess potent antiviral effects against herpes simplex virus (HSV) infection. However, its anti-HSV mechanism has not yet been fully elucidated.

**Purpose:**

This study aimed to investigate the potential mechanisms of PVE30 against HSV infection.

**Methods:**

Antiviral activity was evaluated by a plaque reduction assay, and the EC_50_ value was calculated. Immunofluorescence staining and heparin bead pull-down assays confirmed the interactions between PVE30 and viral glycoproteins. Real-time PCR was conducted to determine the mRNA levels of viral genes, including UL54, UL29, UL27, UL44, and US6, and the proinflammatory cytokines IL-6 and TNF-α. The protein expression of viral proteins (ICP27, ICP8, gB, gC, and gD), the activity of the TLR-NF-κB signalling pathway, and necroptotic-associated proteins were evaluated by Western blotting. The proportion of necroptotic cells was determined by flow cytometric analysis.

**Results:**

The *P. vulgaris* polysaccharide PVE30 was shown to compete with heparan sulfate for interaction with HSV surface glycoprotein B and gC, thus strongly inhibiting HSV attachment to cells. In addition, PVE30 downregulated the expression of IE genes, which subsequently downregulated the expression of E and L viral gene products, and thus effectively restricted the yield of progeny virus. Further investigation confirmed that PVE30 inhibited TLR2 and TLR3 signalling, leading to the effective suppression of NF-κB activation and IL-6 and TNF-α expression levels, and blocked HSV-1-induced necroptosis by reducing HSV-1-induced phosphorylation of MLKL.

**Conclusion:**

Our results demonstrate that the *P. vulgaris* polysaccharide PVE30 is a potent anti-HSV agent that blocks TLR-mediated NF-κB activation.

**Graphical Abstract:**

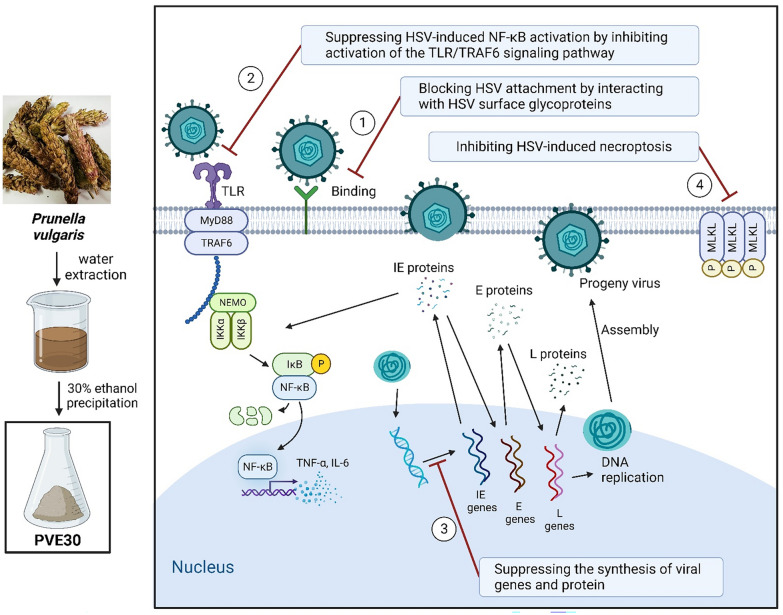

**Supplementary Information:**

The online version contains supplementary material available at 10.1186/s13020-023-00865-y.

## Background

*Prunella vulgaris* is an herbaceous plant belonging to the Labiatae family that is commonly used in traditional Chinese medicine. The compounds and extracts isolated from *P. vulgaris* were reported to possess several potential pharmacological properties, such as antitumour activity and anti-inflammatory, antioxidative, and antiviral effects [1]. Our previous study found that water-extracted *P. vulgaris* exhibited activity against HSV infection [2], and the extract isolated by hot water and 30% ethanol precipitation (PVE30) possessed antiviral effects against not only HSV-1 and HSV-2 but also acyclovir-resistant strains (data unpublished). To further elucidate the antiviral properties of *P*. vulgaris, the molecular mechanism of PVE30 against HSV infection was investigated in this study.

Herpes simplex virus types 1 and 2 (HSV-1, HSV-2) consist of linear double-stranded DNA genomes and belong to the *Alphaherpesvirinae* subfamily. HSVs are prevalent human pathogens; HSV-1 usually causes oropharyngeal infection, and HSV-2 induces genital infection. The initial entry of HSV into cells is mediated by the binding of the virus envelope glycoproteins to the cell surface heparan sulfate (HS) receptor, and targeting this binding interaction is crucial for the development of new antiviral drugs. It has been reported that the deletion of HSV envelope glycoprotein B and gC severely reduced the efficiency of virus attachment and its infectivity [3, 4], and the infection susceptibility was significantly reduced in HS-deficient mutant cells [5, 6].

In addition to blocking viral entry, regulating virus replication is another strategy for the development of anti-HSV drugs. Toll-like receptors (TLRs) play a critical role in controlling HSV infection by recognizing viral proteins and viral nucleic acids. First, as a pathogen-associated molecular pattern (PAMP), HSV glycoprotein B can be recognized by the TLR2 receptor, which then triggers the recruitment of the adaptor molecules myeloid differentiation primary-response protein 88 (MyD88) and tumour necrosis factor receptor-associated factor 6 (TRAF6), leading to the activation of NF-κB [7]. The transcriptional regulator of the NF-κB family plays a key role in regulating host gene expression, which affects cell survival, differentiation, inflammation, and antiviral responses. The persistent activation of NF-κB caused by HSV infection increases the efficiency of virus replication. The translocation of NF-κB failed to occur when virus entry was prevented or when the virus expressed mutated forms of viral ICP4 or ICP27 [8]. Second, TLR3 recognizes the viral dsRNA intermediates generated during HSV-1 replication, and the activation of TLR3 induces receptor-interacting kinase-3 (RIPK3)-dependent necrosis to reduce viral propagation, which is crucial for immune defence against infection [9, 10].

The present study demonstrated that PVE30 inhibited the attachment of HSV to cells by interacting with HSV surface glycoproteins B and gC, thus restricting the yield of progeny virus. In addition, PVE30 effectively suppressed HSV infection by inhibiting TLR-mediated NF-κB activation and blocking HSV-1-induced necroptosis. Our results demonstrate that PVE30 isolated from *P. vulgaris* is a potent antiviral agent that blocks HSV infection.

## Materials and methods

### Preparation of PVE30

Dried spica of *P. vulgaris* L. collected from Henan Province, China was purchased from Shanghai Huaying Pharmaceutical Co. Dried spica of *P. vulgaris* was extracted twice with 20 volumes of purified water for 2 h each time. Ethanol (30%) was added while stirring, and the mixture was stored at 4°. Then, the precipitates were collected and washed with 30% ethanol, named PVE30. It was dissolved in DMEM containing 2% DMSO and stored at − 20 °C until use. The fingerprint spectrum of PVE30 is shown in Additional file 1: Fig. S1.

### Cells, viruses, and antibodies

Vero cells (African green monkey kidney cells; ATCC, USA) were obtained from the American Type Culture Collection (ATCC) and cultured in Dulbecco’s modified Eagle’s medium (DMEM; Gibco, USA) supplemented with 10% fetal bovine serum (Biological Industries, Israel) and 1% antibiotic solution (penicillin‒streptomycin, Gibco, USA). HSV-1 strain KOS and HSV-2 strain G (ATCC, USA) viral stocks were propagated and titrated by plaque-forming unit assay. Antibodies against β-actin (#66,009–1-Ig) were purchased from Proteintech (China). Antibodies against HSV-1/2 ICP27 (sc-69806), HSV-1 ICP8 (sc-53329), HSV-2 ICP8 (sc-56992), HSV-1/2 gB (sc-56987), HSV-1/2 gD (sc-69802), HSV-1 gC (sc-69800), HSV-2 gC (sc-69801) and α-tubulin (sc-5286) were purchased from Santa Cruz Biotechnology (USA). Antibodies against NF-κB p65 (#8242), phospho-NF-κB p65 (#3033), IKKβ (#2678), phospho-IKKα/β (#2697), IκBα (#4812), MyD88 (#4283), and TRAF6 (#8028) were purchased from Cell Signaling Technology (USA). Antibodies against lamin A/C (ab108595), MLKL (ab184718), and phospho-MLKL (ab196436) were purchased from Abcam (UK).

### Plaque reduction assay

After seeding Vero cells (2 × 10^5^ cells/well) on 12-well plates, the cells were cultured overnight at 37 °C with 5% CO_2_. The cell monolayers were prechilled at 4 °C for 30 min and subsequently inoculated with tenfold dilutions of HSV stock at 4 °C for 1 h. After infection, the cells were washed twice with PBS to remove unbound virus and then overlaid with DMEM containing 1% methylcellulose and 2% FBS. After incubation at 37 °C for 72 h, the cell monolayers were fixed in 4% paraformaldehyde solution and subsequently stained with 1% crystal violet. Then, the plaques were counted, and the viral titer was calculated.

### Attachment, penetration, pre-infection, and post-infection assays

Vero cells (2 × 10^5^ cells/well) were seeded and incubated overnight, and then treated with PVE30 at various stages of HSV infection. For the post-infection assay, the cell monolayers were prechilled and subsequently inoculated with 100 PFU/well HSV at 4 °C for 1 h. After infection, the cells were washed twice and then overlaid with DMEM containing 1% methylcellulose, 2% FBS, and various concentrations of extracts or acyclovir (ACV). After incubation at 37 °C for 72 h, the cell monolayers were fixed and stained as described above, and then the plaques were counted. Viral inhibition (%) was calculated as follows: Viral inhibition (%) = [1−(Number of plaques)/(Number of plaques) _control_] × 100. The EC_50_ values were determined by linear regression analysis.

For the attachment assay, the cell monolayers were prechilled and subsequently inoculated with 100 PFU/well HSV in the presence or absence of various concentrations of PVE30 at 4 °C for 1 h. After infection, the cells were treated as described above.

For the penetration assays, the cell monolayers were prechilled and subsequently inoculated with 100 PFU/well HSV. After infection, the cells were washed and treated with various concentrations of PVE30 at 37 °C for 1 h. Then, the cells were treated as described above.

For the pre-infection assays, the cell monolayers were treated with various concentrations of PVE30 at 37 °C for 1 h and subsequently inoculated with 100 PFU/well HSV for 1 h. After infection, the cells were treated as described above.

### Viral inactivation assay

Vero cells (4 × 10^5^ cells/well) were seeded and incubated overnight. HSV (2 × 10^5^ PFU) was incubated with 1 mg/mL PVE30 at 37 °C for 15 min and then diluted 1000-fold prior to infection. The infectivity of the HSV-PVE30 samples was analysed by a plaque reduction assay as described above.

### Pull-down assay

The HSV-1-infected cell lysates were incubated with heparin magnetic beads (Beaverbio, Soochow, China) at 4 ℃ for 1 h to form heparin-bound gB and gC beads. Then, the beads were washed with binding buffer to remove unbound virions. PVE30 (500 μg/mL) was then used to elute the heparin-bound gB or gC at 4 ℃ for 1 h, followed by Western blot assay to detect glycoprotein expression.

### One-step growth curve

Vero cells (4 × 10^5^ cells/well) were seeded and incubated overnight. The cell monolayers were prechilled at 4 °C for 30 min and subsequently inoculated with HSV (MOI = 1). After infection, the wells were washed twice and then treated with DMEM containing 2% FBS supplemented with PVE30 or ACV. The culture media were harvested at 0, 6, 12, 24 and 48 h post infection and stored at − 80 °C. The titer of the infected cells and culture media were measured by a plaque reduction assay as described above.

### Real-time PCR

Cells (4 × 10^5^ cells/well) were seeded and incubated overnight. The cell monolayers were prechilled and subsequently inoculated with HSV at 4 °C for 1 h. After infection, the wells were washed twice and then treated with DMEM containing 2% FBS supplemented with PVE30. Real-time RT-PCR was performed as described previously [11]. The primer sequences are shown in Table 1.

**Table 1:** The primer sequences used in real-time PCR

Genes	Primer sequence (5′-3′)	
Monkey 18S	Forward	ACACGGACAGGATTGACAGATTGATAG
	Reverse	ACCAGACAAATCGCTCCACCAAC
HSV-1 UL54	Forward	TGTCGGGGTCGGAGAGAAGATG
	Reverse	GGCGGTGCGTGTCTAGGATTTC
HSV-2 UL54	Forward	TTGCCTCCTTTGTGTTGGTCATCC
	Reverse	TTTCAATGAGACCCGCCATGCAG
HSV-1 UL29	Forward	AGGCTCGGACAAGGTAACCATAGG
	Reverse	TTGAACGGCTCTGCGATGACAC
HSV-2 UL29	Forward	TGTTTGTGGCGACCGTCAAGAG
	Reverse	CTCAGGTACTCGTCCTCCAGCAG
HSV-1 US6	Forward	ACACCGAATGCTCCTACAACAAGTC
	Reverse	CGCTGAAGCTGTCATAGTAGTTCCAG
HSV-2 US6	Forward	TCTCAGACTCACTCGTGGATGCC
	Reverse	TGATGCCGTCGTAGTAGTTTGTGTG
HSV-1 UL27	Forward	CATATCCACCACCTTCACCACCAAC
	Reverse	GTTGTACCTGCGGGCGAAGATG
HSV-2 UL27	Forward	CACACCACCGACCTCAAGTACAAC
	Reverse	TCGCCAGCACAAACTCATCGTAC
HSV-1 UL44	Forward	TAAAGCCGCCACCCTCTCTTCC
	Reverse	TGCCGTTGTGTTGGTAGGAAAGC
HSV-2 UL44	Forward	TACTGGTGGGTGAACGGAGACG
	Reverse	TCGAAGTTACGAAGCGGACAAACC
HSV-1 UL47	Forward	CCATCACCACGCCCAGTATATCATC
	Reverse	GCCCGCAGATACTCGTTGTTCAG
HSV-2 UL47	Forward	ACCGTCGAGGTTCTATCCATAGTCC
	Reverse	GTAGGTACTCGTTGTTCAGGCTGTC
Human 18S	Forward	GGACACGGACAGGATTGACAGATTG
	Reverse	TAACCAGACAAATCGCTCCACCAAC
Human TNFα	Forward	CCTCATCTACTCCCAGGTCCTCTTC
	Reverse	TCTGGTAGGAGACGGCGATGC
Human IL-6	Forward	ACTCACCTCTTCAGAACGAATTG
	Reverse	CCATCTTTGGAAGGTTCAGGTTG
Human NF-κB	Forward	CCCACGAGCTTGTAGGAAAGG
	Reverse	GGATTCCCAGGTTCTGGAAAC
Human TLR2	Forward	TTGGTCACCGTGGCTCAGAGG
	Reverse	AGCAGAGGTCAGACTTGGGTTAGG
Human TLR3	Forward	CCTTTACTCCTCTGGCGGTTTCG
	Reverse	CTTGGCTGTTCTGGGACTCACTTC
Human TLR4	Forward	GTGAGGATGATGCCAGGATGATGTC
	Reverse	TGACTCCAGCCACATACCTCCAC

### Western blot assay

After treatment as described above, cells were lysed by lysis buffer (40 mL lysis buffer containing 0.25 mL 1.0 M Tris–HCl (pH 6.8), 8 mL 10% SDS and 8 mL glycerol) and boiled for 10 min. Cytoplasmic and nuclear proteins were extracted by a Nuclear and Cytoplasmic Extraction Kit (Beyotime Biotechnology, China). The detailed protocol has been described previously [12]. The bands were quantified by densitometry using ImageJ software and were normalized to the reference control.

### Flow cytometric analysis

After treatment as described above, the HeLa cells were harvested and incubated with an Annexin V-FITC/PI Apoptosis Detection Kit (Meilun Biotechnology, China) and then analysed using CytExpert software.

### Immunofluorescence imaging

Cells (8 × 10^4^ cells/well) were seeded in 12-well plates containing aseptic slides and incubated overnight. After treatment as described above, the wells were washed twice and then fixed in 4% paraformaldehyde solution for 30 min at room temperature. Fixed cells were permeabilized using 0.3% Triton X-100. After blocking with 3% BSA for 1.5 h, the slides were incubated overnight with anti-gB, anti-gC, anti-gD, or anti-phospho-NF-κB p65 antibodies, incubated with a secondary Cy3-conjugated goat antibody for 1 h, and then stained with DAPI (4′,6-diamidino-2-phenylindole) overnight at room temperature. The fluorescence expression of proteins was observed under a fluorescence microscope (Olympus, Japan).

### Statistical Analysis

The results are presented as the mean ± SD values from three independent assays, and statistical significance was determined by GraphPad Prism 8 using one-way ANOVA or Student’s t test. Statistical significance was indicated as *P < 0.05, **P < 0.01, and ***P < 0.001.

## Results

### PVE30 inhibits HSV infection by blocking viral attachment and penetration

**Fig. 1:**
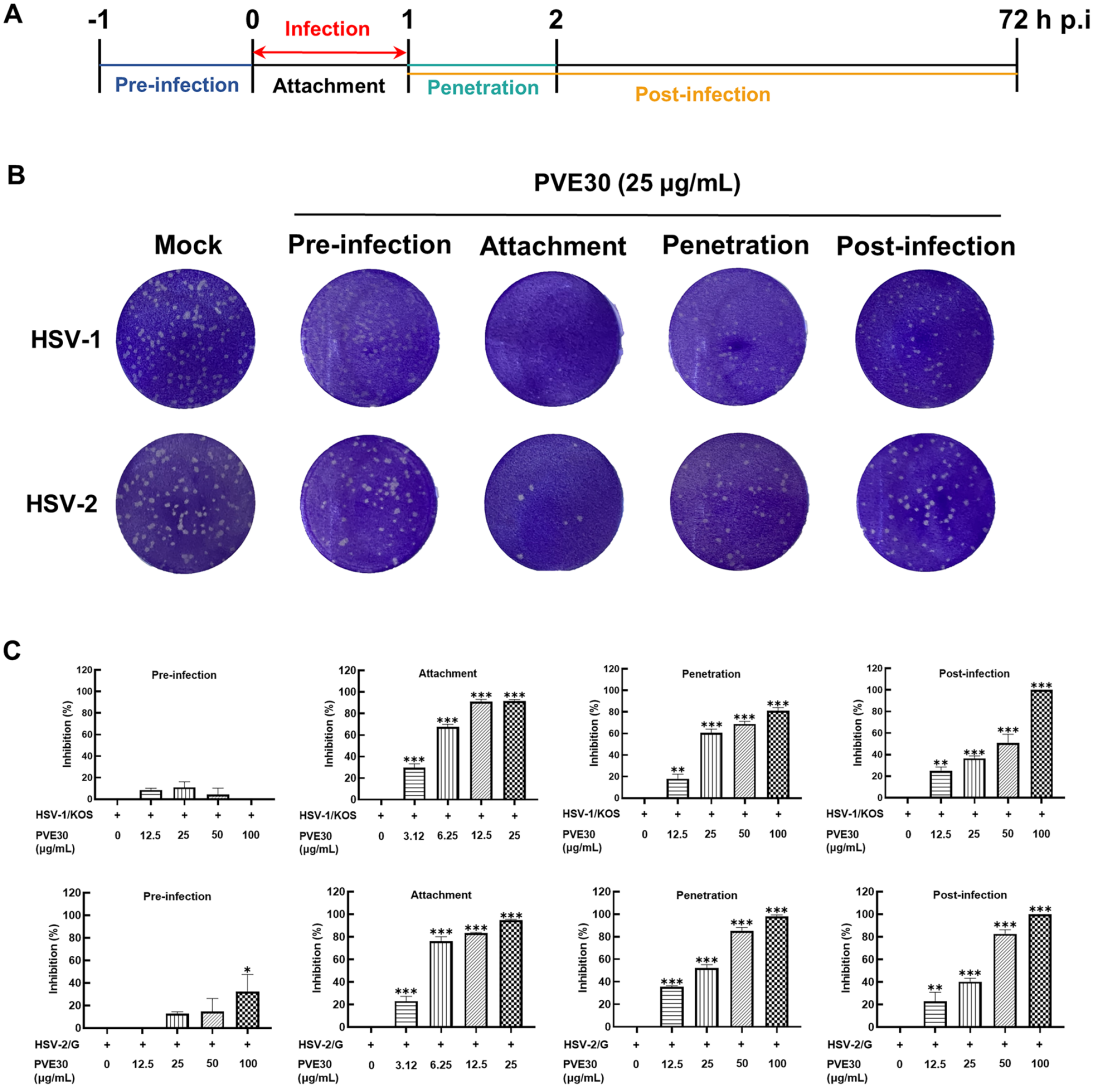
Inhibition effects of PVE30 at different stages of HSV infection in vitro. Vero cells infected with 100 PFU/well HSV-1/KOS or HSV-2/G were treated with PVE30 at various stages of HSV infection, and the EC_50_ values were determined by a plaque reduction assay. (**A**) Simple diagram of PVE30 treatment at different stages of HSV infection. (**B**) Representative images of the plaque reduction assay with PVE30 (25 μg/mL) added at different stages of HSV infection. (**C**) Anti-HSV effects of PVE30 on pre-infection, attachment, penetration and post-infection stage. Values represent the mean ± S.D. from three independent experiments with each treatment performed in duplicate. *** *P* ≤ 0.001, ** *P* ≤ 0.01, * *P* ≤ 0.05, *vs*. virus control group

The antiviral effects of PVE30 and ACV against HSV-1/KOS and HSV-2/G have been verified using the plaque reduction assay (Table 2). To confirm the viral stage targeted by PVE30 during the HSV infection cycle, Vero cells were treated with PVE30 under four different conditions (Fig. 1A): pre-infection (− 1 to 0 h p.i), attachment (0–1 h p.i), penetration (1–2 h p.i), or post-infection (1–72 h p.i) followed by plaque reduction assays. As shown in Fig. 1B and C, the addition of PVE30 during or post-infection significantly reduced HSV infection. Specifically, PVE30 notably reduced HSV attachment to cells, with EC_50_ values of 4.53 ± 0.21 μg/mL for HSV-1/KOS and 4.61 ± 0.40 μg/mL for HSV-2/G (Table 3). Under penetration conditions, PVE30 exhibited a moderate inhibitory effect on virus entry into cells. However, pretreatment of the cells did not influence plaque formation. Thus, it was shown that PVE30 exerts substantial anti-HSV activity, mainly by inhibiting the attachment of the virus to cells and, to a lesser extent, virus penetration into cells, which is different from the mechanism of ACV.

**Table 2:** The anti-HSV EC_50_ values of PVE30 and ACV evaluated by the plaque reduction assay

	HSV-1/KOS	HSV-2/G
PVE30	33.36 ± 0.77	26.61 ± 0.86
ACV	0.10 ± 0.01	0.14 ± 0.01

(Unit: μg/mL)

**Table 3:** The EC_50_ values of PVE30 at different virus infectious cycles evaluated by the plaque reduction assay (Unit: μg/mL)

	HSV-1/KOS	HSV-2/G
Pre-infection	> 100	> 100
Attachment	4.53 ± 0.21***	4.61 ± 0.40***
Penetration	27.86 ± 2.58*	22.77 ± 2.50
Post-infection	33.36 ± 0.77	26.61 ± 0.86

Antiviral effects were evaluated by a plaque reduction assay to determine the 50% effective concentration (EC_50_)

Values represent the mean ± S.D. from three independent experiments with each treatment performed in duplicate

***P < 0.001, *P < 0.05, *vs*. post-infection group

### PVE30 directly targets virions by interacting with HSV surface glycoproteins

**Fig. 2:**
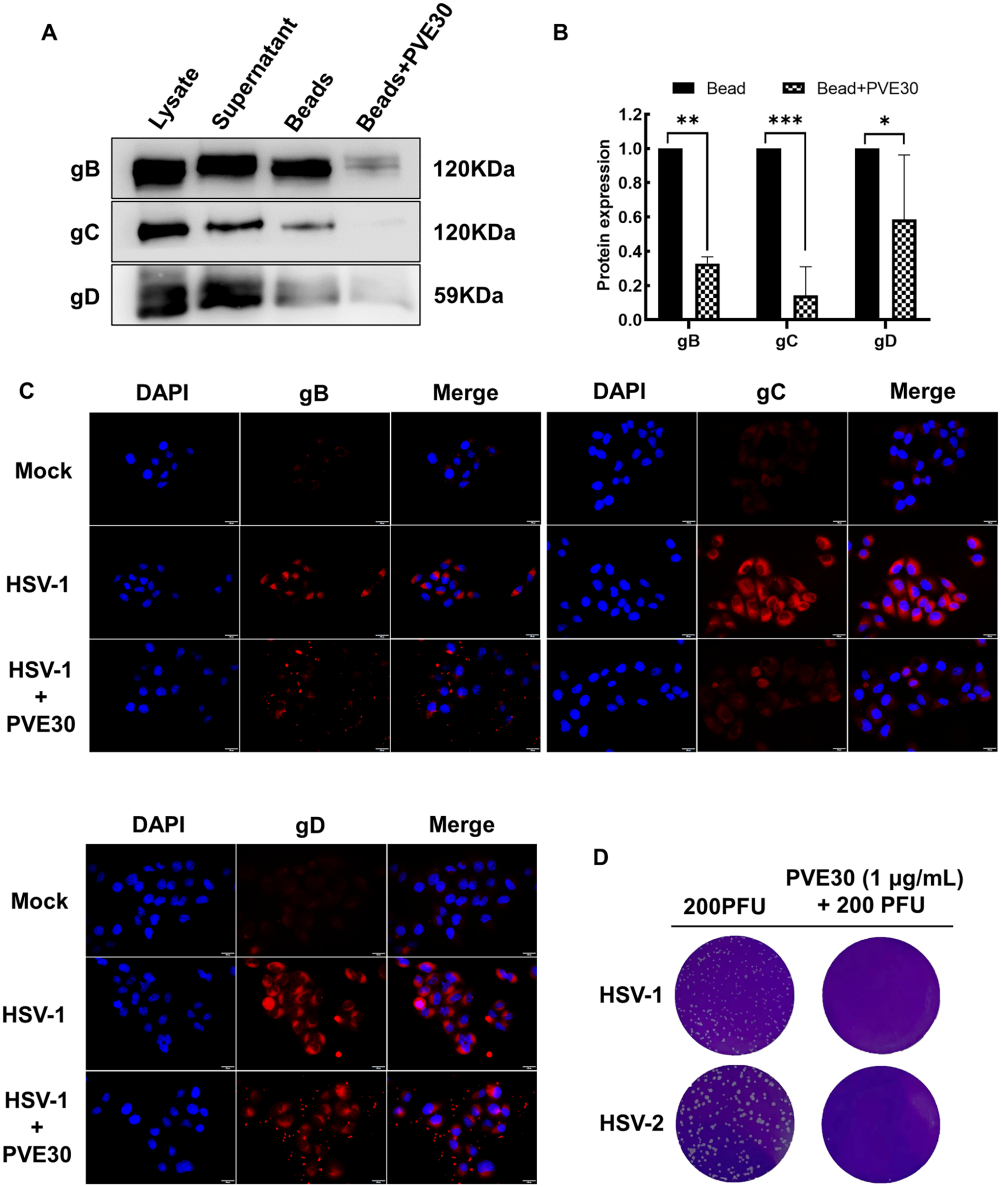
PVE30 inactivated viral particles by interaction with viral glycoproteins. (**A** and **B**) HSV-1-infected cell lysates were incubated with heparin magnetic beads at 4 °C for 1 h, and 500 μg/mL PVE30 was then used to elute heparin-bound gB or gC at 4 °C for 1 h, followed by a Western blot assay to detect gB, gC, and gD expression (n=3). (**C**) HeLa cells infected with HSV-1/KOS at MOI=10 and treated with PVE30 during attachment and penetration. The immunofluorescence expression levels of viral gB, gC and gD were then detected using fluorescence microscopy. Scale bar = 20 μm. (**D**) HSV (2×10^5^ PFU) was incubated with PVE30 (1 mg/mL) at 37 °C for 15 min and then infected into Vero cells at 1000-fold dilution. Infectivity was analysed by a plaque reduction assay (n=3)

Herpesvirus requires binding to particular receptors in order to enter host cells, followed by the coordinated action of multiple viral entry glycoproteins to trigger membrane fusion [13]. Glycoprotein C, gB, and gD mediate the attachment and entry of HSV, with gC and gB binding to HS proteoglycan and gD binding to 3-O-sulfation of HS [14–17]. To investigate whether PVE30 interferes with the interaction between host cell receptors and HSV surface glycoproteins, thus preventing viruses from infecting host cells, a heparin bead pull-down assay was used to evaluate the interactions between heparin and glycoprotein C, gB and gD in the presence of PVE30. As expected, HSV gB, gC, and gD could bind to the heparin magnetic beads, whereas PVE30 blocked virus binding more effectively, as indicated by the faint expression of gB and gC compared to gD (Fig. 2A and B). Consistently, the immunofluorescence expression of gB and gC in the PVE30-treated group was downregulated compared with that in the HSV-1 group (Fig. 2C). In contrast, there was no significant difference in the expression of gD.

To verify the potentially direct effect of PVE30 on virions, HSV was preincubated with PVE30 for 15 min at 37 ℃, and then the mixtures were diluted 1000-fold to infect the cells. The strong reduction corresponded to 99.99% inhibition of HSV-1 plaque formation, supporting that PVE30 directly acted on the viral particles (Fig. 2D). Overall, these results indicate that PVE30 directly targets virions by interacting with HSV surface glycoprotein B and gC.

### PVE30 restricts viral replication by suppressing the mRNA and protein levels of HSV-related genes

**Fig. 3:**
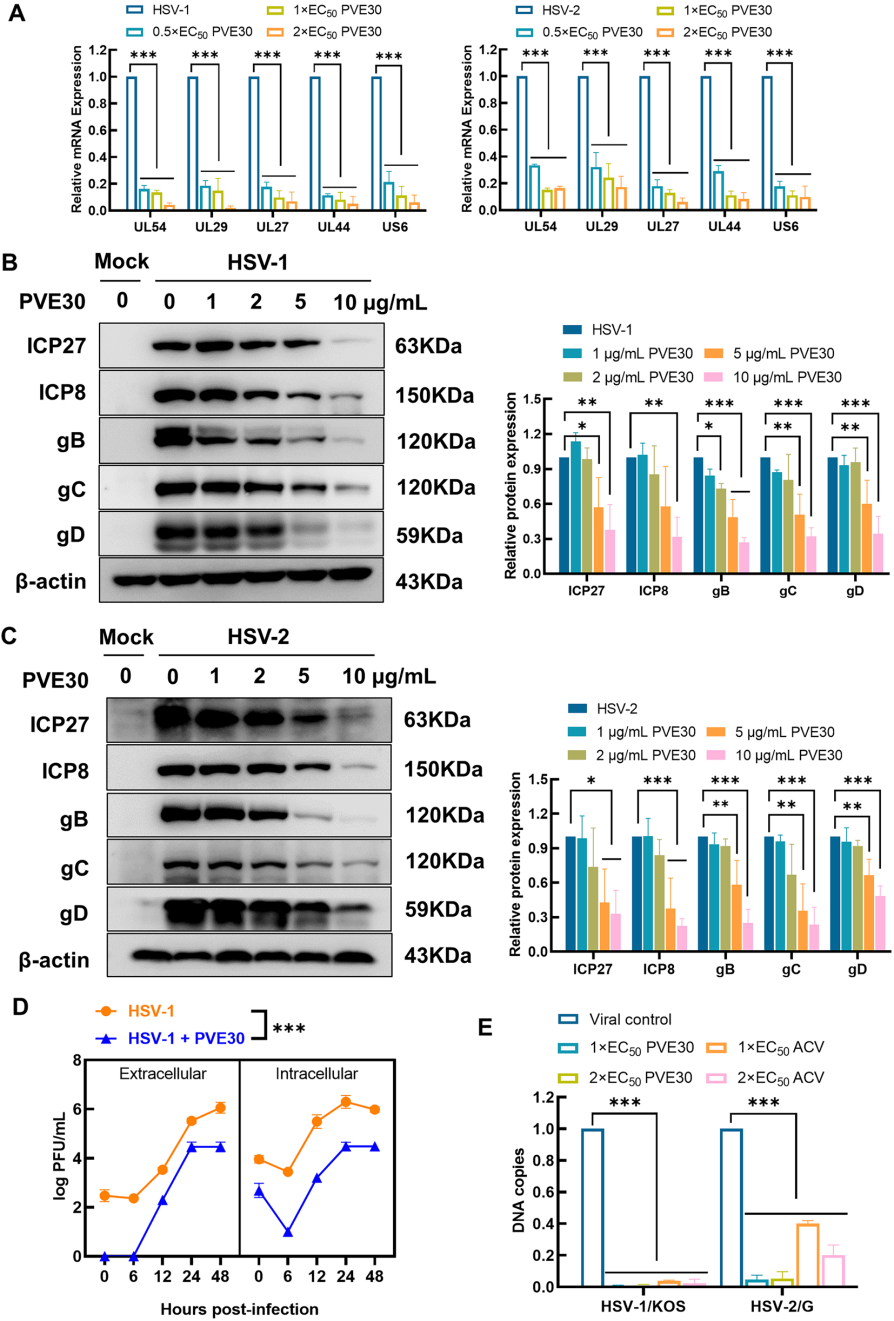
PVE30 suppressed HSV gene and protein expression and inhibits viral replication.  (**A**) Vero cells were infected with HSV-1/KOS or HSV-2/G at an MOI=0.1 for 1 h and treated with or without various concentrations of PVE30 (0.5×EC_50_, 1×EC_50_, and 2×EC_50_). The viral mRNA expression levels (UL54, UL29, UL27, UL44, and US6) were determined at 3, 6, and 9 hours post infection by RT‒PCR (n=3). (**B** and **C**) Vero cells were infected with HSV-1/KOS or HSV-2/G at an MOI=1 for 1 h and treated with or without various concentrations of PVE30 (1, 2, 5, and 10 μg/mL). The viral protein expression levels (ICP27, ICP8, gB, gC, and gD) were determined at 3, 6, and 9 hours post infection by Western blot assay (n=3). (**D**) Vero cells were infected with HSV at an MOI=1 for an hour and treated with PVE30 (50 μg/mL). The infected cells and culture media were harvested at 0, 6, 12, 24 and 48 hours post infection and titered by a plaque reduction assay (n=3). (**E**) Vero cells were infected with HSV-1/KOS or HSV-2/G at an MOI=1 for 1 h and treated with or without various concentrations of PVE30 and ACV (1×EC_50_ and 2×EC_50_). The viral UL47 expression levels were determined at 24 hours post infection by RT‒PCR (n=3). *** *P* ≤ 0.001, ** *P* ≤ 0.01, * *P* ≤ 0.05, *vs*. virus control group

As PVE30 exhibited significant anti-HSV activity against multiple stages of viral infection, we examined the underlying molecular mechanisms of PVE30 on lytic gene expression. The immediate-early (IE) genes play a regulatory role in gene expression, and early (E) genes are involved in viral DNA replication. Most late (L) genes are virion components, and three of them, gB, gC, and gD, are mainly involved in viral entry [18]. The viral mRNA transcriptional levels of UL54 (IE gene), UL29 (E gene), UL27, UL44, and US6 (L gene) were measured by real-time PCR, and their encoded protein expressions of ICP27, ICP8, gB, gC, and gD were analysed by Western blot in Vero cells infected with HSV-1/KOS or HSV-2/G. As shown in Fig. 3A, the mRNA expression levels of the IE, E and L genes were remarkably suppressed after treatment with the indicated concentrations of PVE30 at 3, 6, and 9 h p.i. Consistently, PVE30 dose-dependently decreased the protein levels of viral IE protein (ICP27), E protein (ICP8), and L proteins (gB, gC, and gD) in Vero cells infected with HSV-1/KOS or HSV-2/G (Fig. 3B and C). These findings confirmed that PVE30 mainly inhibited the early stage of viral replication due to the downregulation of IE gene expression and the subsequent attenuation of E and L viral gene products.

To examine the effect of PVE30 on viral replication kinetics, we compared extracellular and intracellular HSV production in the presence or absence of PVE30 using a one-step growth curve assay. As shown in Fig. 3D, both extracellular and intracellular virus efficiently replicated upon HSV-1 infection within 48 h, whereas the addition of PVE30 resulted in a significant reduction in virus titer in the whole period of viral multiplication, and the final yield of the progeny virions was decreased almost 100-fold compared with that of the virus control group. As shown in Fig. 3E, the intracellular viral DNA replication was totally reduced in cells with PVE30 treatment upon infection with HSV-1/KOS and HSV-2/G viruses, which showed equivalent efficacy to that of the positive control ACV. These data demonstrated that PVE30 affects the whole virus replication cycle, not only acting at the early stage of HSV infection but also reducing the yield of progeny virus.

### PVE30 suppressed HSV-induced NF-κB activation

**Fig. 4:**
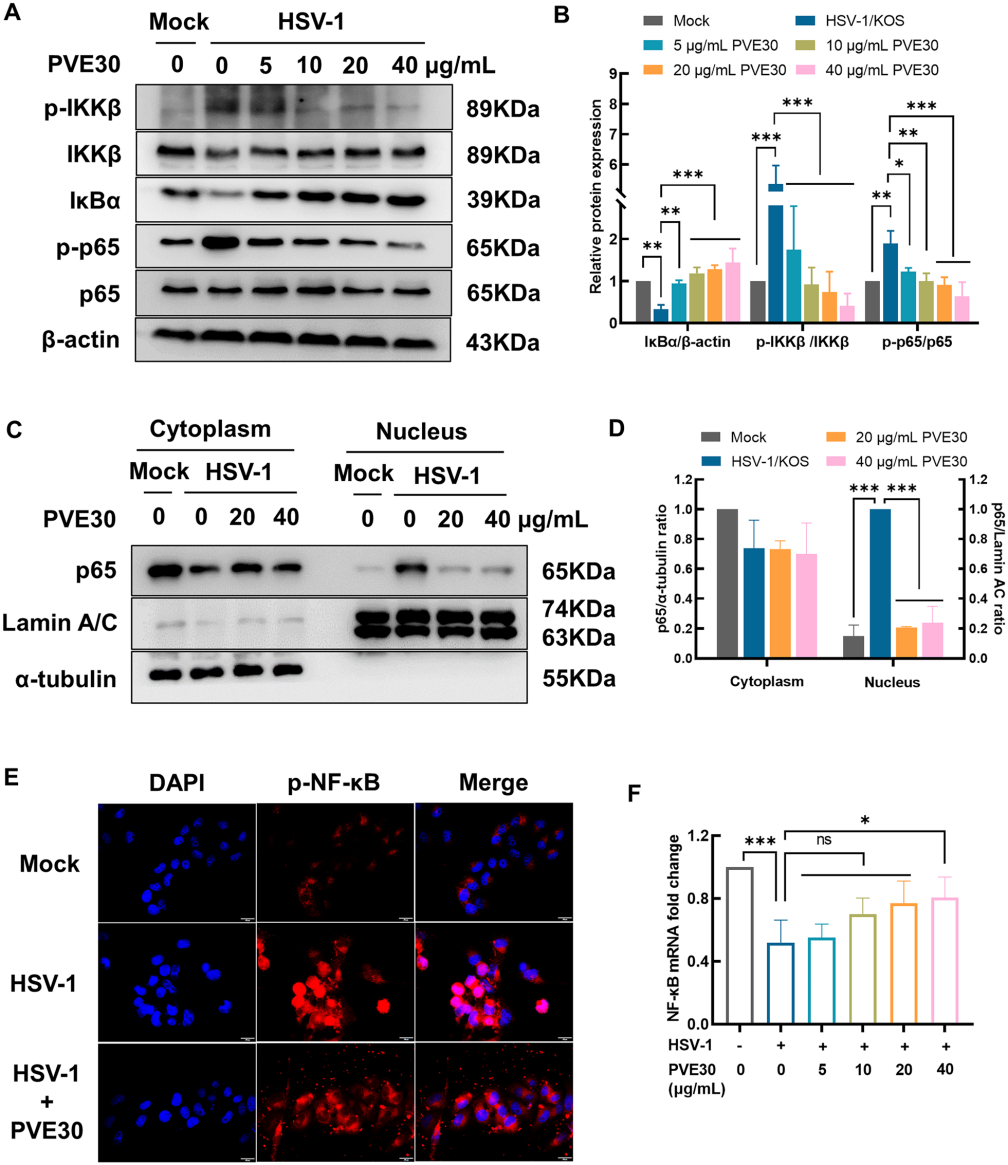
PVE30 suppressed HSV-1-induced NF-κB activation. HeLa cells were infected with HSV-1/KOS at an MOI=1 for 1 h and treated with or without various concentrations of PVE30 (5, 10, 20, and 40 μg/mL) for 24 hours. (**A** and **B**) The protein expression levels (p-IKKβ, IKKβ, IκBα, p-p65, and p65) were determined by Western blot assay (n=3). (**C** and **D**) Cytoplasmic and nuclear proteins were extracted, and p65 nuclear translocation was determined by Western blot assay (n=3). (**E**) HeLa cells were infected with HSV-1/KOS at an MOI=1 for 1 h and treated with PVE30 (40 μg/mL) for 24 hours. The immunofluorescence expression of p-NF-κB was determined by fluorescence microscopy. Scale bar = 20 μm. (**F**) The mRNA expression level of NF-κB was determined by RT‒PCR (n=3). *** *P* ≤ 0.001, ** *P* ≤ 0.01, * *P* ≤ 0.05, *vs*. virus control group

The substantial and persistent NF-κB activation of HSV-1-infected human cells plays an important role in viral pathogenesis and host cell survival [19]. The synthesis of HSV IE proteins was reported to initiate IKK-mediated NF-κB activation with the loss of IκBα and IκBβ, which increases the efficiency of virus replication [8]. To better understand whether PVE30 inhibited HSV-induced NF-κB activation, classical NF-κB signalling and p65 nuclear translocation were evaluated. Compared with the mock group, the HSV-1 post-infection group exhibited increased phosphorylation of IKKβ and p65 and degradation of IκBα, suggesting that persistent NF-κB activation was induced by HSV-1 infection (Fig. 4A and B). PVE30 notably hindered the phosphorylation of IKKβ in a dose-dependent manner without affecting the total protein levels. Furthermore, PVE30 upregulated the protein expression of IκBα and ultimately downregulated the ratio of phosphorylated p-p65 to p65.

NF-B activation is marked by nuclear translocation of NF-B subunits. An examination of cell nuclear and cytoplasmic extracts revealed that HSV infection significantly enhanced the level of NF-B p65 in the nucleus, while treatment with PVE30 suppressed the HSV-1-mediated nuclear translocation of NF-κB p65 (Fig. 4C and D), and similar results were observed by immunofluorescence staining (Fig. 4E). However, the mRNA expression of p65 remained unchanged (Fig. 4F).

### PVE30 blocks NF-κB-mediated proinflammatory cytokine production by inhibiting activation of the TLR/TRAF6 signalling pathway

**Fig. 5:**
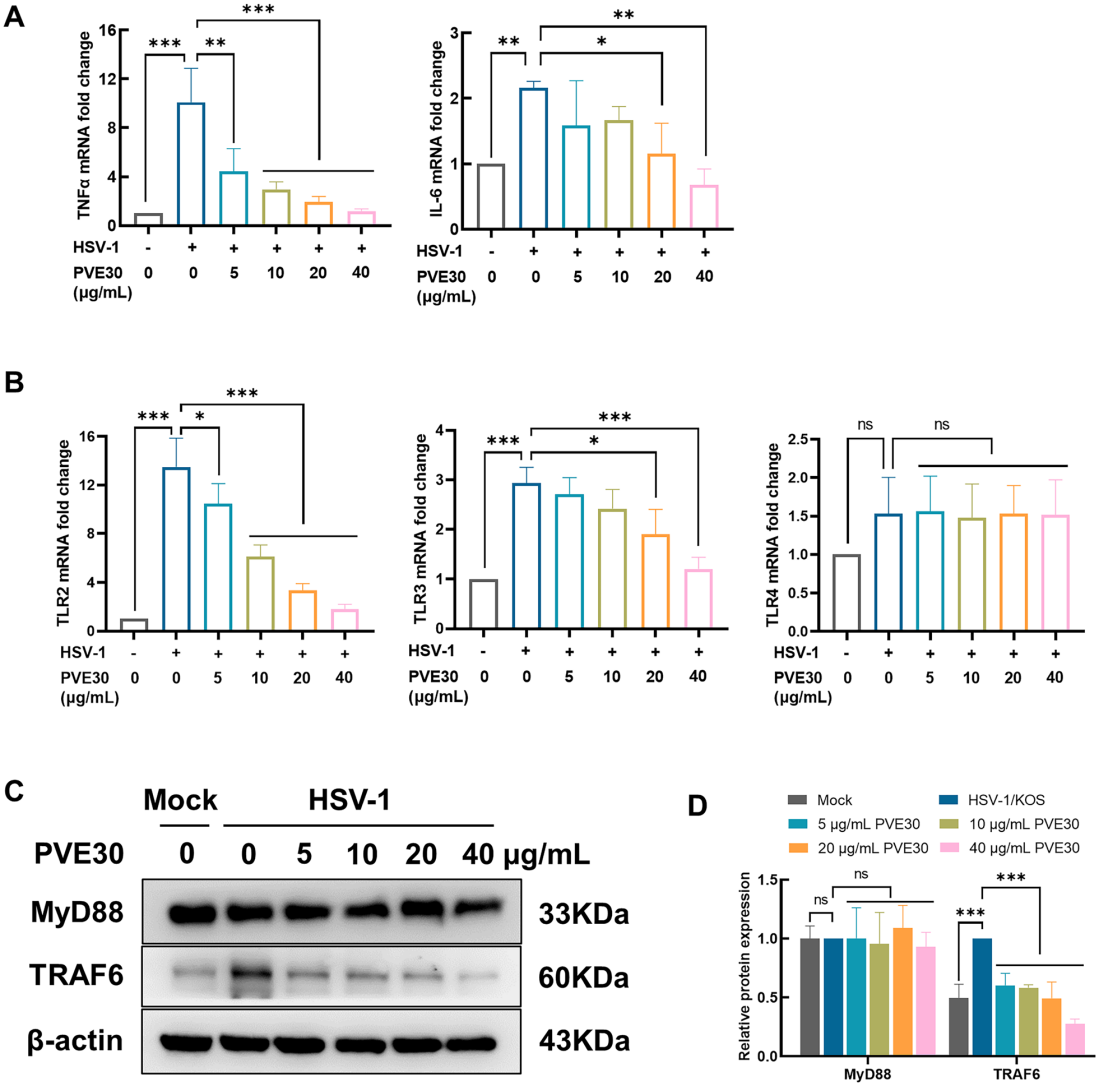
PVE30 suppressed HSV-1-induced activation of TLR pathway. HeLa cells were infected with HSV-1/KOS at an MOI=1 for 1 h and treated with or without various concentrations of PVE30 (5, 10, 20, and 40 μg/mL) for 24 hours. (**A**) The mRNA expression levels of TNFα and IL-6 were determined by RT‒PCR (n=3). (**B**) The mRNA expression levels of TLR2, TLR3, and TLR4 were determined by RT‒PCR (n=3). (**C** and **D**) The protein expression levels of MyD88 and TRAF6 were determined by Western blot assay (n=3). *** *P* ≤ 0.001, ** *P* ≤ 0.01, * *P* ≤ 0.05, *vs*. virus control group

In addition, HSV-infected NF-κB activation upregulates the expression of various inflammatory cytokines, including IL-6 and TNF-α [20, 21]. Coincidentally, the transcriptional expression levels of IL-6 and TNF-α were significantly decreased in PVE30-treated cells infected with HSV-1 (Fig. 5A). TLRs can recognize the specific patterns of microbial components called PAMPs. Human TLRs recognize the structural components of HSV pathogens and transmit signals through MyD88 or TRAF6, which trigger NF-κB activation and promote the release of inflammatory cytokines and chemokines [9]. To examine the possibility that PVE30 inhibits NF-κB through TLR signalling, the mRNA level of TLRs and the protein expression of MyD88 and TRAF6 were evaluated. As shown in Fig. 5B, HSV-1 infection increased the mRNA levels of TLR2 and TLR3, with a slight change in TLR4. In contrast, PVE30 significantly decreased the mRNA levels of TLR2 and TLR3, which indicated that PVE30 alleviated HSV-1-activated TLR signalling in HeLa cells. Furthermore, PVE30 reduced the protein level of TRAF6 expression without any obvious trend in MyD88 expression (Fig. 5C and D). Overall, PVE30 inhibited TLR2 and TLR3 signalling through the downregulation of TRAF6 and blocked HSV-1-induced NF-κB activation to interfere with viral replication.

### PVE30 prevents HSV-1-induced necroptosis

**Fig. 6:**
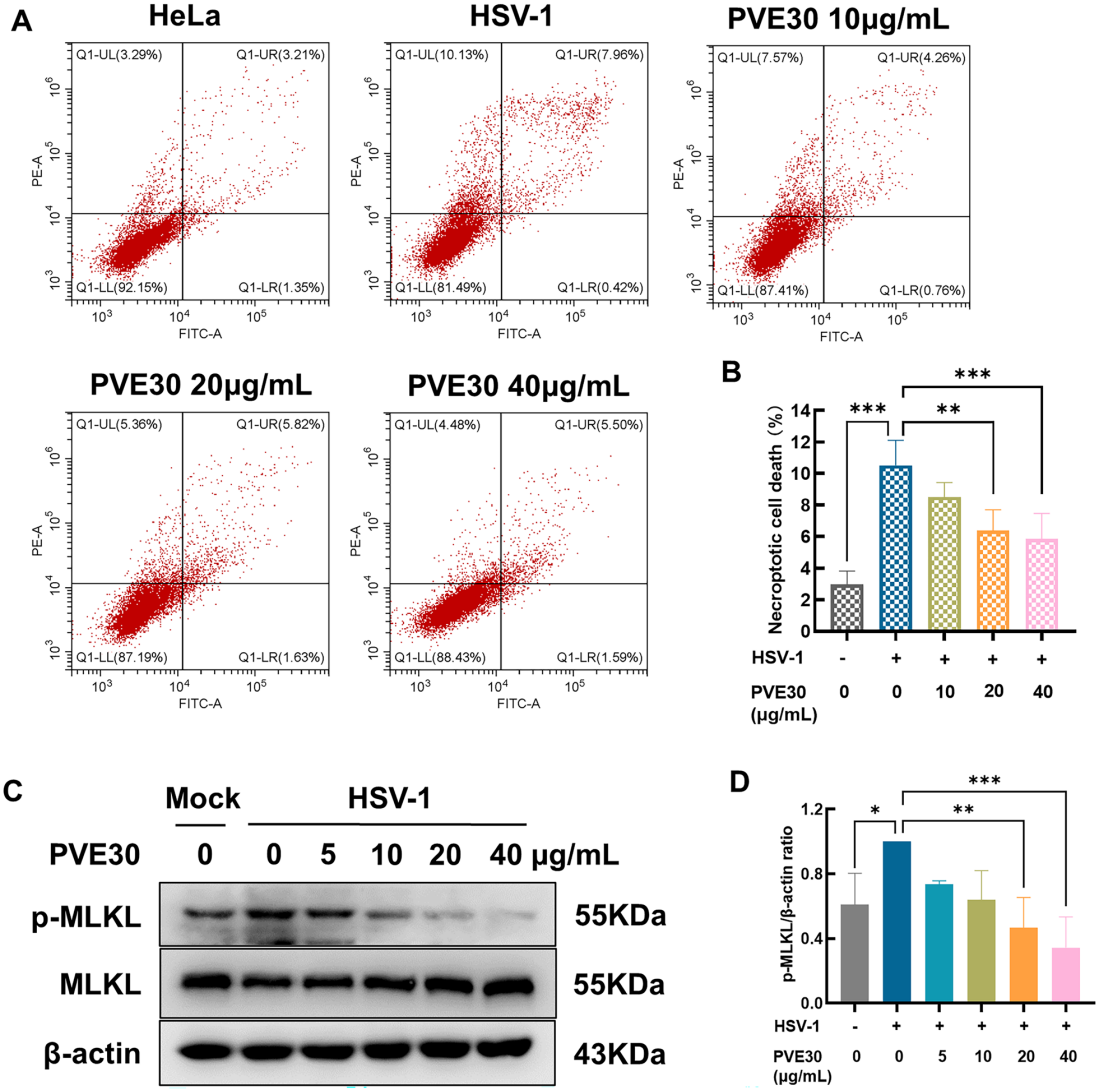
PVE30 suppressed HSV-1-induced necroptosis. HeLa cells were infected with HSV-1/KOS at an MOI=1 for 1 h and treated with or without various concentrations of PVE30 (10, 20, and 40 μg/mL) for 24 hours. (**A** and **B**) The cells were harvested and incubated with an Annexin V-FITC/PI Apoptosis Detection Kit and then analysed using CytExpert software. LL (lower left) represents viable cells (%), UL (upper left) represents necroptotic cells (%), LR (lower right) represents early apoptotic cells (%), UR (upper right) represents late apoptotic cells (%), (n=3). (**C** and **D**) The protein expression levels of p-MLKL and MLKL were determined by Western blot assay (n=3). *** *P* ≤ 0.001, ** *P* ≤ 0.01, * *P* ≤ 0.05, *vs*. virus control group

Necroptosis has also been implicated in microbe infection-associated death. Viral infection can be sensed by TLRs, which have been proven to activate programmed necrosis [22]. To clarify whether PVE30 inhibits HSV-induced necroptosis, flow cytometric analysis with an Annexin V/Propidium Iodide (PI) assay was performed. As shown in Fig. 6A and B, HSV-1 infection increased the percentage of cells stained with PI, indicating the induction of necroptosis. Notably, PVE30 decreased the percentage of HSV-1-induced necroptosis. As a hallmark of necroptosis, the protein expression of MLKL was evaluated. As shown in Fig. 6C and D, the phosphorylation of MLKL accumulated after HSV-1 infection, which was effectively reversed by PVE30 in a dose-dependent manner. These data suggested that PVE30 prevented HSV-induced necroptosis.

## Discussion

Naturally occurring sulfated polysaccharides possess a broad-spectrum antiviral effect and interfere with virus adsorption, invasion, uncoating, transcription, replication, assembly and release steps in the virus life cycle [23]. Our previous study suggested that the *Prunella vulgaris* polysaccharide extracted by hot water may inhibit HSV by competing for cell receptors as well as by several unknown mechanisms after the virus has penetrated the cells [2]. In the present study, PVE30 was reported to possess antiviral activities against HSV-1 and HSV-2 with EC_50_ values of 33.36 ± 0.77 μg/mL and 26.61 ± 0.86 μg/mL, respectively, at the post-infection stage. In addition, PVE30 exhibited more efficient antiherpetic activities in the attachment stage, that is, PVE30 inhibited virus particles attaching to the host cell with EC_50_ values of 4.53 ± 0.21 μg/mL and 4.61 ± 0.40 μg/mL, respectively (Table 3). Herein, this study focused on deciphering the mechanism by which PVE30 inhibits viral attachment and viral replication.

HS, a cellular glycosaminoglycan (GAG) chain of heparan sulfate proteoglycans (HSPGs) [24], participates in the attachment and entry steps of certain viruses [25], including HSV [15], human immunodeficiency virus type 1 (HIV-1) [26], and SARS-CoV-2 [27]. In the case of HSV, HS serves as a receptor in the initial attachment step, binding to virion surface glycoprotein C and gB, thus mediating the HSV invasion process [15]. A heparin bead pull-down assay was performed to investigate whether the inhibitory effect of PVE30 on viral attachment was related to interfering the interaction between HS receptors and gB or gC. Heparin and heparan sulfate are two structurally related analogues, and the pull-down assays indicated that virus gB and gC proteins could bind to heparin-coupled magnetic beads and that PVE30 treatment impaired their interaction (Fig. 2A and B). In addition, the PVE30-treated HSV particles were barely infective (Fig. 2D), suggesting that PVE30 directly inactivates virions by interacting with HSV surface gB and gC.

In addition, the mechanism by which PVE30 restricts viral replication was further investigated. PVE30 downregulated the expression of the IE gene UL54 and its product ICP27 protein (Fig. 3A–C). ICP27 is essential for lytic infection, which mainly inhibits precursor mRNA splicing and promotes nuclear export of viral transcripts at the posttranscriptional level [18, 28]. Thus, the attenuation of E and L viral gene product expression after PVE30 treatment was attributed to the downregulation of IE genes (Fig. 3B and C). PVE30 affects the entire virus replication cycle and reduces the yield of progeny virus (Fig. 3D and E).

The activation of NF-κB was found to prolong the survival of host cells to facilitate replication and increase viral progeny production [19, 29]. The first wave of NF-κB activation is triggered rapidly by the binding of gD to herpesvirus entry mediator A (HveA). After 3–4 h of infection, the synthesis of IE viral proteins leads to the second wave of NF-κB pathway activation, which is substantial and persistent [19]. Herein, the role of NF-κB signalling was investigated in the presence of PVE30 upon HSV infection. PVE30 alleviated HSV-1-triggered NF-κB activation by inhibiting the IKKβ phosphorylation, upregulating the protein expression of IκBα, and downregulating the protein expression and nuclear translocation of p65 (Fig. 4A and C). In addition, PVE30 reduced the secretion of the uncontrolled downstream inflammatory cytokines IL-6 and TNF-α from host cells upon infection with HSV-1 (Fig. 5A). It has also been reported that HSV infection activates NF-κB signalling through TLRs, thus leading to the production of cytokines [9]. For HSV-1, TLR2 recognizes glycoprotein B and gH/gL to initiate the activation of NF-κB and mediates the induction of proinflammatory cytokines [7, 30]. TLR3 also activates NF-κB to upregulate IL-6 and TNF-α in HSV-1-infected astrocytes [31]. Our study demonstrated that PVE30 may inhibit TLR2 and TLR3 signalling through the suppression of HSV-1-induced TRAF6-mediated NF-κB activation, thus interfering with viral replication (Fig. 5B and C).

The balance between cell death, proliferation and differentiation is crucial for microbe infection-associated death [22]. During HSV-1 replication, viral dsRNA intermediates can be sensed by TLR3, which leads to necroptosis by forming the RIPK3/TRIF complex [10, 32]. In this study, we clarified that PVE30 decreased the percentage of HSV-1-induced necroptosis and the protein expression levels of p-MLKL (Fig. 6A and C), suggesting that PVE30 also inhibited HSV-induced necroptosis. Given that the protein level of MyD88 expression remains unaltered following HSV-1 infection, viral infection leads to an elevation in the mRNA levels of TLR2 and TLR3, an effect that can be mitigated by PVE30 (Fig. 5B and C). Consequently, it is plausible that PVE30 inhibits HSV-induced necroptosis through the TLR3 signalling pathway, which is recognized as a MyD88-independent TLR signalling pathway.

**Fig. 7:**
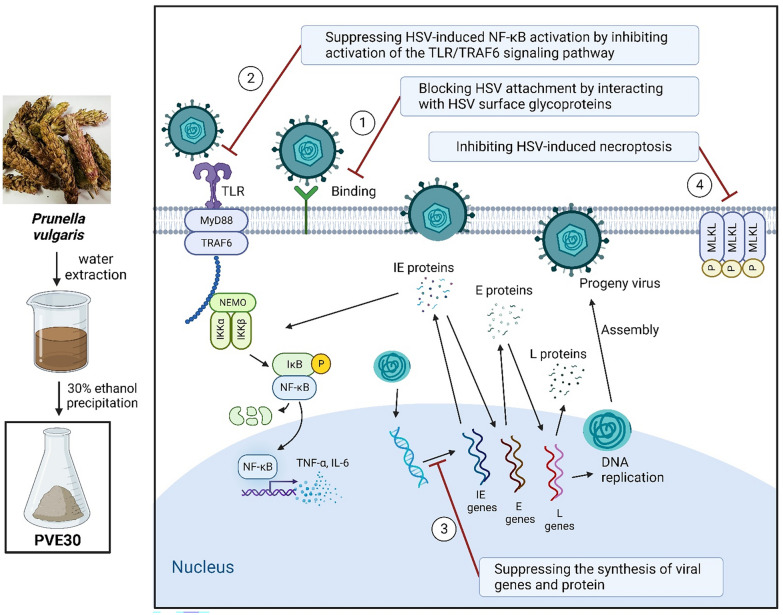
A schematic of the mechanism of PVE30 in inhibiting HSV

In conclusion, our study demonstrated that PVE30 is a potent antiviral agent against HSV infection. PVE30 inhibited the attachment of HSV by competing with HS and inactivated virions. In addition, PVE30 effectively restricted viral replication via the inhibition of NF-κB and TLR signalling and prevented HSV-1-induced necroptosis (see Fig. 7).

## Conclusion

Our study demonstrated that the *P. vulgaris* polysaccharide PVE30 mainly inhibited HSV infection by directly inactivating virions and restricting viral replication. Mechanistically, PVE30 inactivated the TLR/TRAF6-mediated NF-κB signalling pathway and inhibited HSV-1-induced necroptosis. Overall, PVE30 has multiple mechanisms of action that make it a promising antiviral agent for HSV infections.

### Supplementary Information

**Additional file 1: Figure S1.** The fingerprint spectrum of PVE30.

## Data Availability

All data generated or analysed during this study are included in this published article [and its Additional information files].

## Abbreviations

ACV: Acyclovir
DMEM: Dulbecco’s Modified Eagle Medium
DMSO: Dimethyl sulfoxide
FBS: Fetal bovine serum
GAG: Glycosaminoglycan
HIV-1: Human immunodeficiency virus type 1
HS: Heparan sulfate
HSPGs: Heparan sulfate proteoglycans
HSV: Herpes simplex virus
HveA: Herpesvirus entry mediator A
IL: Interleukin
MLKL: Mixed-lineage kinase domain-like protein
MyD88: Myeloid differentiation primary-response protein 88
NF-κB: Nuclear factor kappa-light-chain-enhancer of activated B cells
PAMPs: Pathogen-associated molecular patterns
PBS: Phosphate-buffered saline
PFU: Plaque forming unit
PI: Propidium Iodide
PVE30: 30% Ethanol precipitation of Prunella vulgaris (L.)
RIPK3: Receptor-interacting protein kinase 3
TLRs: Toll-like receptors
TNF: Tumour necrosis factor
TRAF6: Tumour necrosis factor receptor-associated factor 6
