# Supplementary figures and images for: Prunella vulgaris polysaccharide inhibits herpes simplex virus infection by blocking TLR-mediated NF-κB activation
Source: Chin Med. 2024 Jan 8;19:6. doi: 10.1186/s13020-023-00865-y (PMC10773030; doi:10.1186/s13020-023-00865-y)

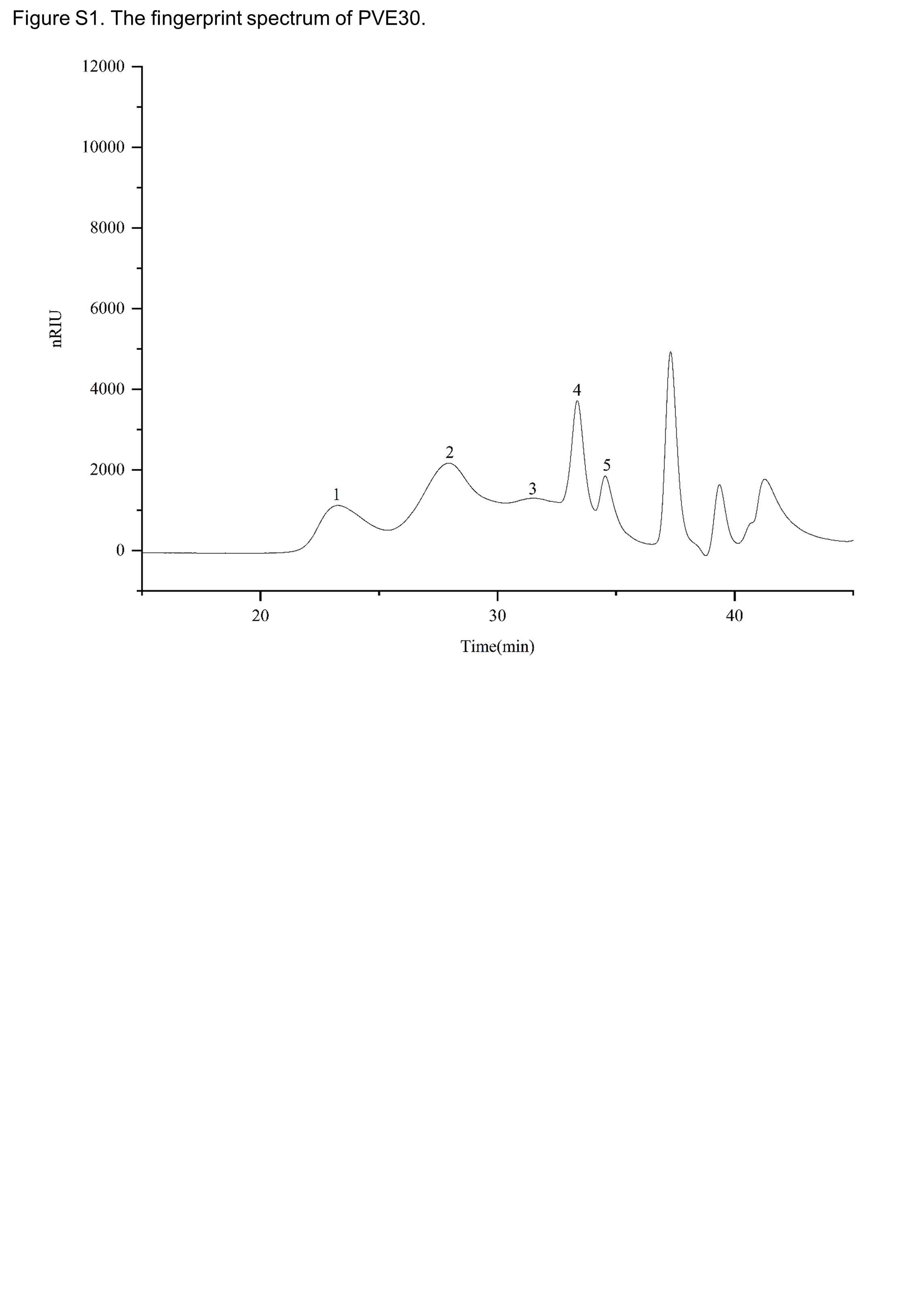

Supplement: Supplementary file 1 — Additional file 1: Figure S1. The fingerprint spectrum of PVE30. [file 13020_2023_865_MOESM1_ESM.tif]
